# Detection of Novel Visible-Light Region Absorbance Peaks in the Urine after Alkalization in Patients with Alkaptonuria

**DOI:** 10.1371/journal.pone.0086606

**Published:** 2014-01-23

**Authors:** Yasunori Tokuhara, Kenichi Shukuya, Masami Tanaka, Mariko Mouri, Ryunosuke Ohkawa, Midori Fujishiro, Tomoo Takahashi, Shigeo Okubo, Hiromitsu Yokota, Makoto Kurano, Hitoshi Ikeda, Seiji Yamaguchi, Shinobu Inagaki, Mika Ishige-Wada, Hiromi Usui, Yutaka Yatomi, Tatsuo Shimosawa

**Affiliations:** 1 Department of Clinical Laboratory, The University of Tokyo Hospital, Tokyo, Japan; 2 The Group of Neurobiology, Division of Health Sciences, Graduate School of Medicine, Osaka University, Osaka, Japan; 3 Department of Diabetes and Metabolic Diseases, Graduate School of Medicine, The University of Tokyo, Tokyo, Japan; 4 Department of Pediatrics, Shimane University Faculty of Medicine, Izumo, Japan; 5 Department of Clinical Laboratory Medicine, Graduate School of Medicine, The University of Tokyo, Tokyo, Japan; 6 Department of Pediatrics and Child Health, Nihon University School of Medicine, Tokyo, Japan; Imperial College London, United Kingdom

## Abstract

**Background:**

Alkaptonuria, caused by a deficiency of homogentisate 1,2-dioxygenase, results in the accumulation of homogentisic acid (2,5-dihydroxyphenylacetic acid, HGA) in the urine. Alkaptonuria is suspected when the urine changes color after it is left to stand at room temperature for several hours to days; oxidation of homogentisic acid to benzoquinone acetic acid underlies this color change, which is accelerated by the addition of alkali. In an attempt to develop a facile screening test for alkaptonuria, we added alkali to urine samples obtained from patients with alkaptonuria and measured the absorbance spectra in the visible light region.

**Methods:**

We evaluated the characteristics of the absorption spectra of urine samples obtained from patients with alkaptonuria (n = 2) and compared them with those of urine specimens obtained from healthy volunteers (n = 5) and patients with phenylketonuria (n = 3), and also of synthetic homogentisic acid solution after alkalization. Alkalization of the urine samples and HGA solution was carried out by the addition of NaOH, KOH or NH4OH. The sample solutions were incubated at room temperature for 1 min, followed by measurement of the absorption spectra.

**Results:**

Addition of alkali to alkaptonuric urine yielded characteristic absorption peaks at 406 nm and 430 nm; an identical result was obtained from HGA solution after alkalization. The absorbance values at both 406 nm and 430 nm increased in a time-dependent manner. In addition, the absorbance values at these peaks were greater in strongly alkaline samples (NaOH- KOH-added) as compared with those in weakly alkaline samples (NH4OH-added). In addition, the peaks disappeared following the addition of ascorbic acid to the samples.

**Conclusions:**

We found two characteristic peaks at 406 nm and 430 nm in both alkaptonuric urine and HGA solution after alkalization. This new quick and easy method may pave the way for the development of an easy method for the diagnosis of alkaptonuria.

## Introduction

Alkaptonuria is a rare (1/100,000 to 1/250,000) and hereditary metabolic disorder characterized by absence of the enzyme homogentisic acid (HGA) oxidase. This defect leads to the accumulation of HGA, a product of phenylalanine and tyrosine metabolism [1]. Urine of patients with alkaptonuria contains HGA in excessive amounts (1–8 g/day) [2] and turns black in color when left to stand owing to the oxidation of HGA to benzoquinone acetic acid (BQA), which in turn is converted with time to a melanin-like pigment. This color change is accelerated when the urine pH is alkaline. Dark brown pigmentation is also observed in the connective tissues, skin, sclera and ear of patients with alkaptonuria [3], [4]. This accumulation of HGA and its metabolites in the tissues causes ochronosis, characterized by darkening of the cartilaginous tissues and bone, which may lead to arthritis, joint destruction, and deterioration of the cardiac valves [1], [4]–[6].

The classical method for the diagnosis of alkaptonuria is to observe the change in color of urine after it is allowed to stand at room temperature for a few days. Recently, diagnosis has become possible by a gas chromatography-mass spectrometry analysis method developed for the detection of HGA in the urine. In addition, genetic analysis has also been conducted for the diagnosis of alkaptonuria. While alkaptonuria is definitively associated with deficient homogentisate 1,2-dioxygenase (HGD) activity in the liver [7], and only mutations of the HGD gene are known to cause alkaptonuria [8]–[10], the aforementioned methods are very expensive and time-consuming.

In the present study, in an attempt to establish a quick, easy and versatile method for the diagnosis of alkaptonuria, we evaluated the characteristics of the absorption spectra of the urine of patients with alkaptonuria and compared them with those of HGA solution after alkalization. We found novel and characteristic peaks in both, which we consider may pave the way for the development of a simple screening test for alkaptonuria.

## Materials and Methods

### Patients

Two alkaptonuria patients were recruited for this study. Case #1 was diagnosed at the University of Tokyo Hospital and was a 61-year-old man with osteoarthritis of the knee. The clinical diagnosis of alkaptonuria was made based on the detection of dark brown pigmentation of the cartilage and connective tissues during surgery for osteoarthritis of the knee. The diagnosis of alkaptonuria was confirmed by the detection of HGA in the urine by gas chromatography mass spectrometry (GC/MS). The urinary organic acid component showed a large fraction of homogentisic acid (HGA).

Case #2 was selected from pooled cases of alkaptonuria at Shimane University. Owing to the use of the urine samples of these patients for research purposes, the privacy of the case is protected and the precise history is blinded, however, the diagnosis was confirmed by GC/MS.

Three cases of phenylketonuria (22-year-old male and 23- and 29-year old females) were recruited from Nihon University Hospital. In all three cases, the diagnosis was made by newborn screening and confirmed by observation of elevated serum phenylalanine levels during the neonatal period. Tetrahydrobiopterin (BH4) deficiency was ruled out by the BH4 loading test. All the patients were under treatment with a low-protein diet, with phenylalanine-free milk. However, their diet adherence was inadequate and the serum phenylalanine levels at the time of the urine sampling for this study were 995, 796 and 603 µmol/L respectively.

### Urine Samples

Urine samples were collected from the patients with alkaptonuria (Case #1 and Case #2), patients with phenylketonuria (Case 1, 2 and 3) and healthy volunteers (5 men). Written informed consent from the patients was obtained for the use of the urine samples. The study was conducted with the approval of the Institutional Research Ethics Committee of the Faculty of Medicine, the University of Tokyo.

### Reagents

Sodium hydroxide (NaOH), potassium hydroxide (KOH), aqueous ammonia (NH4OH), gentisic acid (2,5-dihydroxybenzoic acid), and ascorbic acid (AA) were purchased from Wako Pure Chemical Industries, Ltd. (Osaka, Japan). HGA was obtained from MP Biomedicals, LLC (Illkirch, France) and 2-hydroxyphenylacetic acid from Sigma-Aldrich Co. (St. Louis, MO).

### Transient Spectrum Measurement

The sample solutions were measured in a model U-3310 spectrophotometer (Hitachi High-Technology Co., Ltd., Tokyo, Japan) with microcells having a path length of 10 mm. For alkalization, 12.5 µL of 1 M NaOH, 1 M KOH or 1 M aqueous NH4OH was added to 750 µL of the urine samples or 750 µL of 0.04% HGA solution in distilled water. The sample solutions were incubated at room temperature for 1 min, 10 min, 30 min, and 60 min prior to the measurements. To confirm the effects of an antioxidant, 150 µL of 1 M AA was added to 600 µL of the urine samples or 600 µL of 0.04% HGA, followed by the addition of 12.5 µL of 1 M NaOH. The sample solutions were then incubated at room temperature for 1 min, and then their absorption spectra measured. For each assay, the urine from Case #1 of alkaptonuria was used without dilution, while that from Case #2 was diluted 8 times with distilled water. The urine samples from the patients with phenylketonuria were used without dilution for each assay. Furthermore, 750 µL of 0.04% 2-hydroxyphenylacetate or 0.04% gentisic acid solution in distilled water was incubated with 12.5 µL of 1 M NaOH for 1 min and the absorption spectrum was measured. All the measurements of the absorption spectra were made with a 1-nm bandwidth at a scan speed of 100 nm/min, with distilled water set as the blank. The absorbance values at 406 nm and 430 nm were measured every 10 seconds on two separate analytical runs.

## Results

### Absorption Spectra in the Visible Region

**Figure 1:**
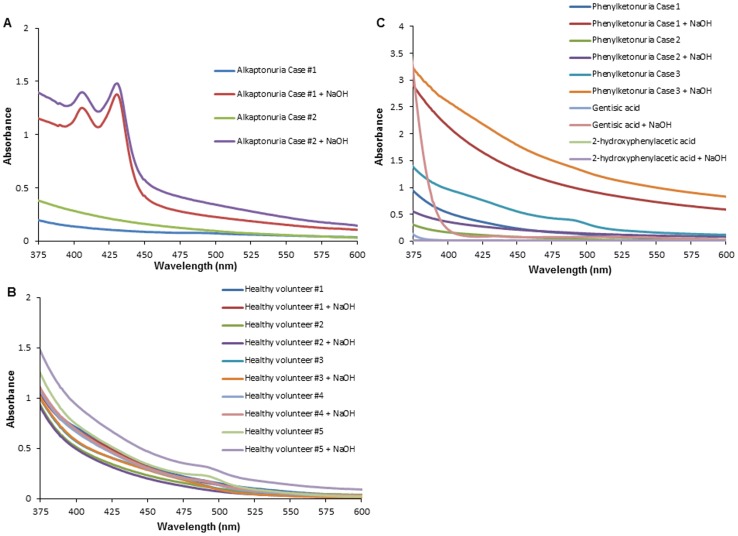
Absorption spectra of alkaptonuric urine showed characteristic peaks following the addition of NaOH. The absorption spectra of urine samples from the patients with alkaptonuria (Case #1 and Case #2) after treatment with 1 M NaOH for 1 min, B, The absorption spectra of urine samples from healthy individuals (#1–#5) after incubation with 1 M NaOH at room temperature for 1 min. C, The absorption spectra of urine samples from patients with phenylketonuria (Cases 1, 2 and 3), and solutions of 0.04% gentisic acid and 0.04% 2-hydroxyphenylacetic acid after treatment with 1 M NaOH for 1 min.

**Figure 2:**
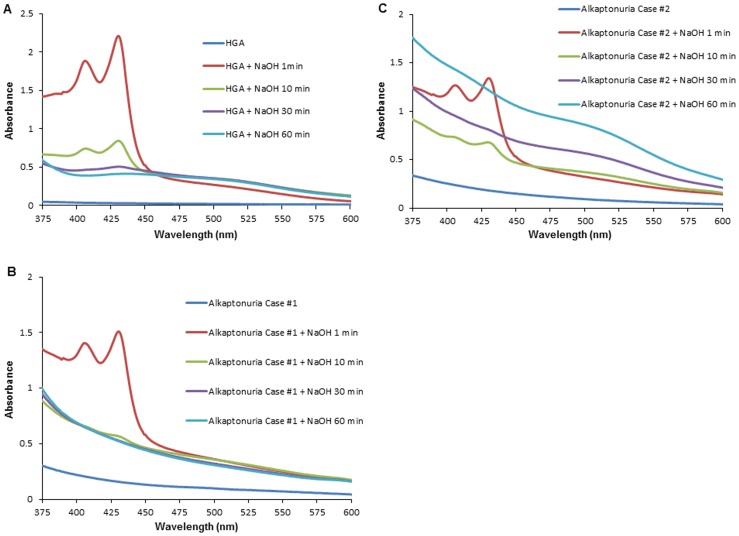
The absorbance conversion of HGA and alkaptonuric urine after addition of NaOH. The absorption spectra of 0.04% HGA solution (A) and the urine samples from Case #1 (B) and Case #2 (C) of alkaptonuria incubated with NaOH at room temperature for each of the indicated durations (1 min, 10 min, 30 min, and 60 min).

**Figure 3:**
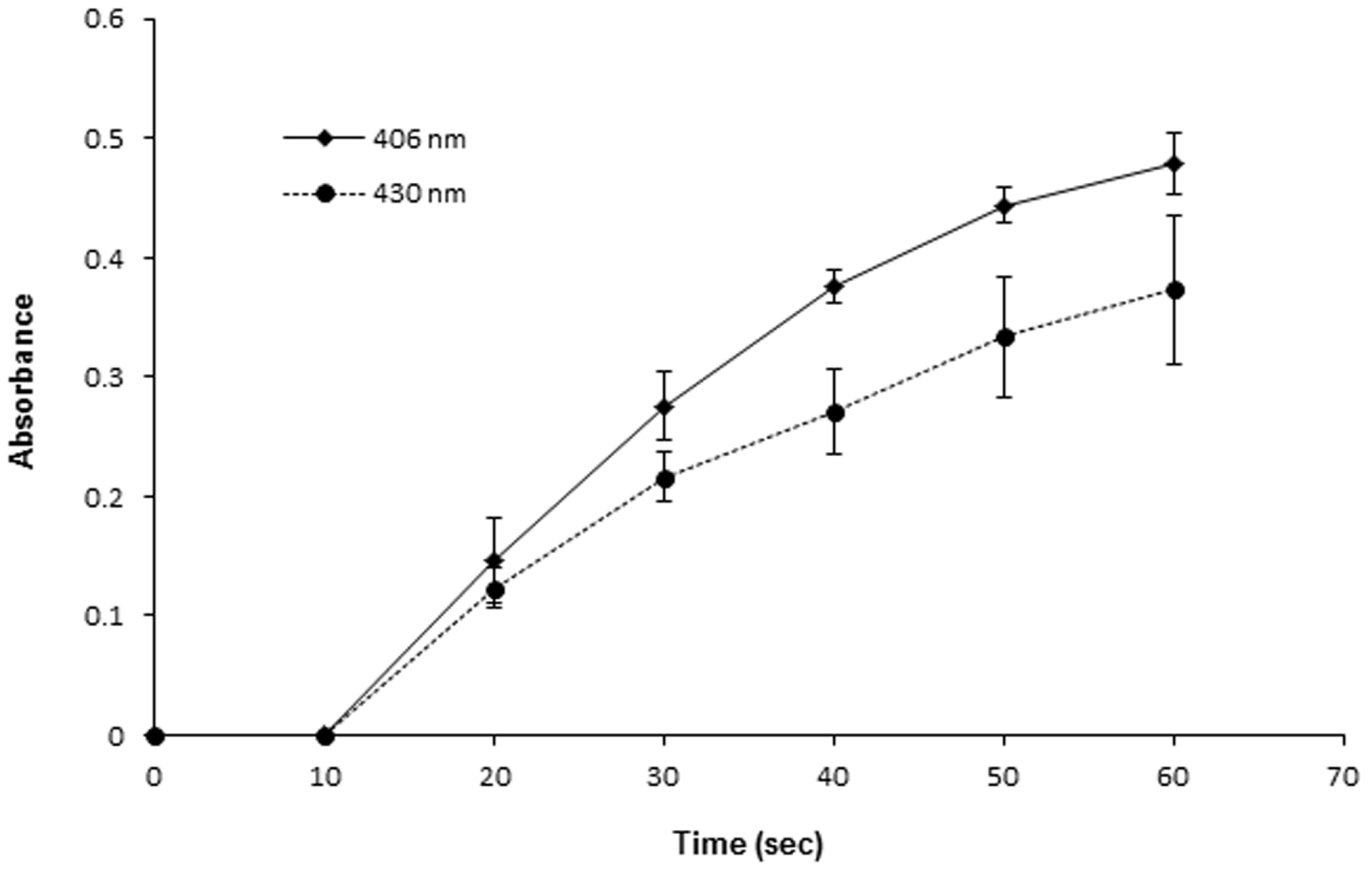
The absorbance values at 406 nm and 430 nm increased in a time-dependent manner. Absorbance increase at 406(♦) and 430 nm (•) of 0.04% HGA solution in the presence of 1 M NaOH. Results are the mean ± S.E. of three experiments.

First, we conducted spectrophotometric analysis at 375–600 nm to detect the absorption curve of the urine samples in the visible light region. The absorption spectra of the urine samples from the alkaptonuria patients incubated with NaOH showed two characteristic peaks at 406 nm and 430 nm (Figure 1A), while no peaks were observed in the absence of NaOH addition. On the other hand, the absorption curve of the urine from healthy individuals, patients with phenylketonuria and gentisic acid solution did not show the peaks at 406 nm and 430 nm, regardless of whether NaOH was added or not (Figure 1B and 1C). The spectrum of the HGA solution also showed characteristic peaks at 406 nm and 430 nm after the addition of NaOH (Figure 2A), comparable to the absorption spectra of the urine samples from the patients with alkaptonuria. Then, we examined the time-course of the spectral profile of the HGA solution and alkaptonuric urine after the addition of NaOH (Figure 2B and 2C). The absorption spectrum of HGA solution showed two sharp peaks after incubation with NaOH for 1 min, however, the two peaks decreased in size after 10 min incubation, and disappeared altogether after 60 min incubation (Figure 2A). Similar results were obtained for alkaptonuric urine (Figure 2B and 2C). With increase of the incubation time of the HGA solution with NaOH up to 1 minute, the absorbance values at 406 nm and 430 nm also increased in size in a time-dependent manner (Figure 3).

### Incubation with Several Alkaline Solutions

**Figure 4:**
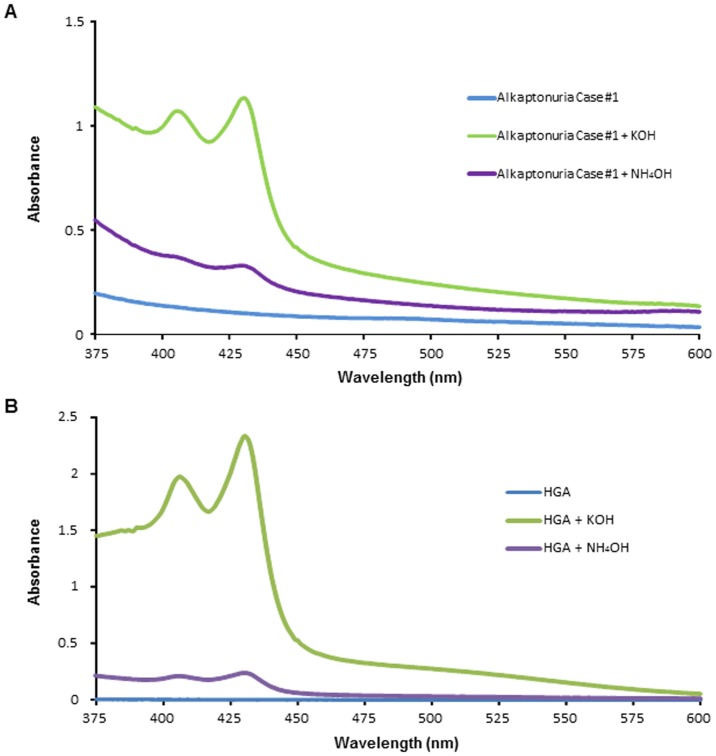
Absorption spectra of the urine samples incubated with KOH or NH4OH showed two peaks. The absorption spectra of the urine sample from a patient with alkaptonuria Case #1 (A) and 0.04% HGA solution (B) after incubation with 1 M KOH or 1M NH4OH at room temperature for 1 min.

Next, we examined the absorption spectra obtained following the addition of other alkaline solutions (KOH, NH4OH) to the urine samples or HGA solution. The spectra of the urine samples of the alkaptonuria patients incubated with KOH or NH4OH at room temperature for 1 min showed two peaks at 406 nm and 430 nm (Figure 4A). Similar results were obtained for the HGA solution (Figure 4B). Furthermore, both for the patient urine and HGA solution, the absorbance values at 406 nm and 430 nm were higher following the addition of KOH than following the addition of NH4OH (Figure 4A and 4B).

### Effect of Ascorbic Acid

**Figure 5:**
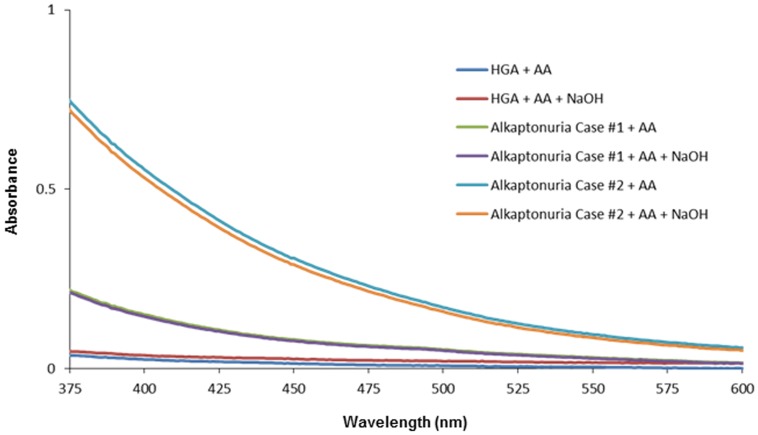
Effects of AA on the absorption spectra of HGA and alkaptonuric urine incubated with NaOH. 1% HGA solutions or the urine samples from the patients with alkaptonuria (Case #1 and Case #2); then, 1 M NaOH was added to each sample, followed by incubation of the samples at room temperature for 1 min.

To inhibit the oxidation of HGA to BQA, we used AA as the antioxidant. A high-dose of AA has been reported to decrease the urinary BQA, while having no effect on the excretion of HGA [11], and treatment with an excess of AA has been reported to reverse the oxidation of HGA [12]. The spectrum of the HGA solution treated with AA showed no peaks at 406 nm and 430 nm following NaOH addition (Figure 5). Similar results were obtained for the alkaptonuric urine samples treated with AA (Figure 5). These results reflect that the two peaks at 406 nm and 430 nm develop gradually with progression of the oxidation of HGA to BQA.

## Discussion

In this study, we attempted to develop a new screening test for alkaptonuria based on measurement of the urine absorption spectra. Our investigation revealed that both alkaptonuric urine and HGA solution showed characteristic absorption spectra with peaks at 406 nm and 430 nm that appeared one minute after alkalization and lasted for less than 60 minutes (Figure 2). In addition, the absorbance values at these peaks were higher in the urine samples that were more strongly alkalized as compared to that in those that were more weakly alkalized. These results are expected to pave the way for the development of a new rapid method for determining HGA in the urine, and these distinct peaks at 406 nm and 430 nm observed following alkalization of the urine may be important elements in a simple screening test for alkaptonuria.

A number of spectrophotometric studies of urine samples from alkaptonuria patients have been conducted in the ultraviolet (UV) region. This is because HGA has been reported to show characteristic absorption at 290 nm [12]. Moreover, with the oxidation of HGA, a second peak gradually begins to appear at 250 nm [13]. This peak is identical to that corresponding to benzoquinone acetic acid (BQA), which is formed by the oxidation of HGA [12], [14]. The oxidation of HGA to BQA is accelerated by the addition of alkali [13], [15]. HGA is slowly oxidized to BQA in the presence of atmospheric oxygen or air, and further oxidized to a more complex polymer with an absorption spectrum different from the spectra of naturally occurring melanins [13]. The pigment deposited in the tissues of alkaptonuric patients with ochronosis has not been analyzed by the same method [16], therefore, the relevance of the model polymerization to the in vivo process has not yet been established [17].

Alkaptonuria is diagnosed based on detection of a change in the color of urine after it is left to stand for several hours to days at room temperature. Alkalization can accelerate the change in color, however, this has not yet been standardized. Currently, definitive diagnosis can be obtained by genetic analysis [8]–[10] or quantification of the urinary HGA by mass spectrometry, both of which are very expensive, time-consuming, and available only at limited facilities.

To save the time and the cost for the current diagnosis method, recently spectrum studies of HGA have been conducted in the UV region from 200 nm to 300 nm. It is reported that the UV spectrum of HGA contains a single peak at 290 nm [12]. The same result was obtained for the urine sample from our patients with alkaptonuria (data not shown). Furthermore, we confirmed the absorption spectra in the UV region of 0.04% HGA and urine from a patient with alkaptonuria after alkalization with 1 M NaOH, and observed a progressive increase of the absorbance value at 250 nm (data not shown). In addition to this UV range shift, we found the new feature of the absorbance peaks of HGA at 406 nm and 430 nm after alkalization of the sample. In order to examine the specificity of this peak, we also examined urine samples from phenylketonuria patients and solutions of 2-hydroxyphenlacetic acid and gentisic acid (2,5-dihydroxybenzoic acid), a downstream metabolite of aspirin [18], because HGA, 2,5-hydroxyphenylacetate, is very similar in structure to both 2-hydroxyphenylacetate and gentisic acid. The urine 2-hydroxyphenylacetate concentration is increased in patients with phenylketonuria [19]–[22]: due to severe hyperphenylalaninemia, phenylalanine is metabolized via a by-path to phenylpyruvate, 2-hydroxyphenylacetate, phenyllactate, and phenylacetate. The urine from patients with phenylketonuria has been reported to turn dark green color following the addition of ferric chloride (FeCl3) [23], [24]. We confirmed color change of the urine in the samples from the patients with phenylketonuria, which were converted to a dark green color after the addition of FeCl3 (data not shown). However, the absorption curve of the urine from the patients with phenylketonuria did not show peaks at either 406 nm or 430 nm, regardless of whether NaOH was added or not (Figure 1C). Moreover, 2-hydroxyphenylacetic acid showed almost no absorbance in the visible light region, with or without the addition of NaOH (Figure 1C). Also, the absorption curve of gentisic acid did not show any peak at 406 nm or 430 nm after the addition of NaOH (Figure 1C). These data suggest that this current new method is specific for HGA and applicable to the diagnosis of alkaptonuria.

The absorbance values at the two peaks (406 nm, 430 nm) of alkaptonuric urine samples and HGA solution after incubation with an alkaline solution increased as the pH increased. This result indicates that HGA was oxidized to BQA in a pH-dependent fashion. This oxidation was rapid, since two sharp peaks were observed even at 1 min after the addition of NaOH or KOH and disappeared within about 60 minutes (Figure 2). As HGA is highly reactive with oxygen to form BQA, which is a semi-quinone or quinone [25], two peaks 406 nm and 430 nm are observed. However, most previous reports do not allude to these peaks; this could be attributable to the examinations of the absorbance being performed several hours after the oxidation in these studies. Menon et al. reported that 16 hours of oxidation of HGA solution or alkaptonuric urine results in the formation of melanin-like pigments which show no absorbance peaks at either 406 nm or 430 nm [26]. The chemical characteristics of these pigments resemble those of pheomelanin or eumelanin [27], [28], although they still remain to be precisely characterized [29]. HGA is non-enzymatically oxidized first to its quinone analog, BQA, and then to a more complex polymer [12], [13], [30]. Under alkalization, such as by the addition of NaOH, HGA shows a marked increase in oxygen utilization, far in excess of that produced by autoxidation or by enzymatic preparation [15]. Rapid oxidation of HGA after alkalization, which represents non-enzymatic oxidation, appeared to induce progressive increase of the absorbance at 406 nm and 430 nm.

As this method is dependent on HGA oxidation-dependent, the appearance of the two peaks at 406 nm and 430 nm and the color change of alkaptonuric urine after alkalization were blunted by the addition of AA because of interference by AA of the oxidation of HGA to BQA (Figure 5). In a previous study also, addition of AA was reported to significantly reduce the levels of BQA in the urine [11]. Protein restriction and treatment with AA may reduce the plasma HGA levels [31]. Therefore, our new method may yield false-negative results in alkaptonuric patients on high-dose AA or a strict protein-restricted diet.

In the present study, we measured the absorbance from the early phase to the late phase and propose that our finding offers a promising rationale for the development of a quick and easy screening test for alkaptonuria.

In summary, we developed a new method to measure the urine absorbance spectrum in the visible light region after alkalization and propose that this simple, cost-effective, versatile and quick method may pave the way for the development of an easy method for the diagnosis of alkaptonuria.
